# Gamma-Hemolysin Components: Computational Strategies for LukF-Hlg2 Dimer Reconstruction on a Model Membrane

**DOI:** 10.3390/ijms24087113

**Published:** 2023-04-12

**Authors:** Costanza Paternoster, Thomas Tarenzi, Raffaello Potestio, Gianluca Lattanzi

**Affiliations:** 1 Department of Physics, University of Trento, Via Sommarive 14, I-38123 Trento, Italy; costanza.paternoster@unitn.it (C.P.);; 2 INFN-TIFPA, Trento Institute for Fundamental Physics and Applications, Via Sommarive 14, I-38123 Trento, Italy

**Keywords:** gamma-hemolysin, pore-forming toxins, membrane proteins, molecular dynamics

## Abstract

The gamma-hemolysin protein is one of the most common pore-forming toxins expressed by the pathogenic bacterium *Staphylococcus aureus*. The toxin is used by the pathogen to escape the immune system of the host organism, by assembling into octameric transmembrane pores on the surface of the target immune cell and leading to its death by leakage or apoptosis. Despite the high potential risks associated with *Staphylococcus aureus* infections and the urgent need for new treatments, several aspects of the pore-formation process from gamma-hemolysin are still unclear. These include the identification of the interactions between the individual monomers that lead to the formation of a dimer on the cell membrane, which represents the unit for further oligomerization. Here, we employed a combination of all-atom explicit solvent molecular dynamics simulations and protein–protein docking to determine the stabilizing contacts that guide the formation of a functional dimer. The simulations and the molecular modeling reveal the importance of the flexibility of specific protein domains, in particular the N-terminus, to drive the formation of the correct dimerization interface through functional contacts between the monomers. The results obtained are compared with the experimental data available in the literature.

## 1. Introduction

Pore-forming toxins (PFTs) are a class of virulence agents secreted by bacteria to escape the immune system of the host organism [1,2,3]. Released as soluble monomers, PFTs later bind and assemble on the membrane of the target cells, undergoing a conformational change that leads to the formation of a transmembrane structure, from which lipids escape; the uncontrolled flux of water, ions, and small molecules through the pore eventually leads to the death of the cell by leakage or apoptosis. PFTs play a major role in the pathogenesis of several bacterial strains, showing increasing resistance toward antibiotics, such as the methicillin-resistant *Staphylococcus aureus*, identified by the World Health Organization as one of the pathogens requiring urgent development of new treatments [4]. Unveiling the steps leading to pore formation from *S. aureus* PFTs can be instrumental for the structure-based development of new antivirulence agents [1]; for instance, the recognition of membrane receptors, the identification of the residues at the protomer interfaces that are crucial for oligomerization, and the analysis of the conformational rearrangements that are required for pore formation may enable the structure-based design of drugs that prevent the toxicity of a given PFT. This is the case of oroxylin A, oroxin A, and oroxin B, which have been reported to bind to the monomeric form of the PFT α-hemolysin and to inhibit its hemolytic activity by preventing the transition toward the lytic pore complex [5,6].

**Figure 1:**
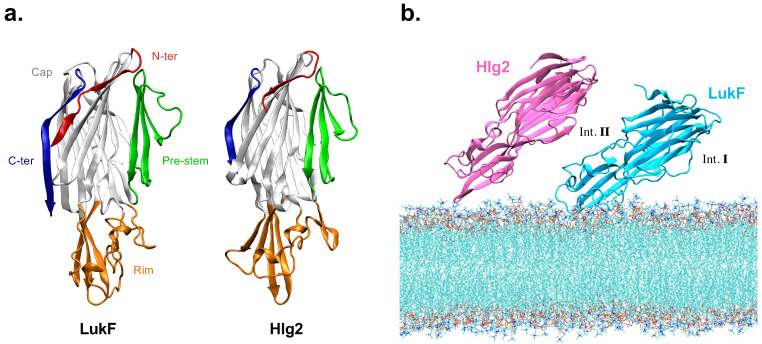
(**a**) Graphical representation of LukF and Hlg2 structures in the monomeric form. Different colors are used to distinguish the functional domains. (**b**) Starting configuration employed in the simulations. Hlg2 is placed in proximity of interface II (int. II) of LukF, the one exposing the N-ter, at a distance larger than the cutoff used for the non-bonded interactions.

The bi-component γ-hemolysin (γ-HL) toxin is one of the most common PFTs expressed in the vast majority of human *S. aureus* isolates [7]. It is composed of two distinct proteins, namely LukF (also called HlgB) and Hlg2 (also called HlgA), which are assembled in an alternate fashion to form an octameric pore. Despite a sequence identity as low as 30%, the two components share very similar structural features (Figure 1). Both proteins are composed of a large-cap domain, namely the central β-sandwich structure where most of the inter-protomer interactions are established in the assembled pore; a rim domain, located beneath the cap domain, which interacts with the lipids of the membrane; and a prestem domain domain, which in the monomeric form of the toxin is folded into a three-stranded antiparallel β-sheet and is anchored to the upper part of the cap by hydrogen bonds and to the N-terminus by hydrophobic interactions. According to the suggested mechanism of pore formation [8], each water-soluble monomeric component binds to the target cell membrane through the rim domain, forms a ring-shaped oligomeric complex, and releases the prestem domain from the upper part of the cap; following oligomerization, the prestem unfolds, and subsequently refolds as a two β-strand stem spanning the whole bilayer, to cooperatively form a transmembrane β-barrel structure that includes the stem domains of all the other protomers. Structural experimental data are available for the LukF monomer [9], the prepore structure [8], and the final pore [10]; in addition, the recent observation was made of a supramolecular assembly of octameric pore complexes formed after membrane lysis [11], whose structure has been resolved by cryo-electron microscopy. The only available experimental structure of a LukF-Hlg2 dimer corresponds to an engineered covalent complex, where the two proteins are linked by a disulfide bridge [12]; the resulting heterodimer is nonetheless able to form pores with similar features to the wild type toxin. The relative orientation of the proteins in the crystallographic model, however, does not match the low-resolution shape of the heterodimer in a solution constructed starting from small-angle X-ray scattering (SAXS) measurements [12]. In the former, the two subunits are oriented at an angle of almost 120 degrees, which is much larger than the angle between the axes of nearby subunits in the assembled octamer (corresponding to approximately 30 degrees); conversely, SAXS data suggests the presence of a variety of orientations, with a more parallel orientation prevailing in a solution. Taken together, these results suggest a certain plasticity of the contacts between the two proteins, which might be required for a step-by-step formation of a functional interface. Two observations suggest that some local rearrangements are required during the formation of the dimer: first, the 15-residue-long N-terminal domain of LukF (N-ter, also called the amino-latch) is folded in the structure of the monomer, but unfolded in the pore [10]. Second, the same contact interface between the proteins, as observed in the final pore, is not possible due to the steric hindrance between the folded prestem domains, meaning that the protomers can readjust to form the final interface observed in the pore only after the release of the prestem; at the same time, the presence of a folded prestem does not impair oligomerization [13].

The adjustments required at the protein interfaces during the dimerization stage make it difficult to predict the steps leading to the structure of a LukF-Hlg2 dimer directly from the individual components. In this work, we tackle this problem by integrating molecular modeling and molecular dynamics (MD) simulations to study the evolution of the contacts between the two proteins, highlighting the role of the N-terminal region. MD simulations proved to be valuable tools for the study of the PFTs from different organisms [14,15,16,17,18,19,20]; in the present work, unbiased simulations were performed, starting from the initial configuration in which the LukF component is anchored to the membrane, while the Hlg2 protein is in a solution above the membrane. Recently, indeed, results from molecular dynamics simulations [21] were integrated with previously published experimental data [22,23] to shed light on the different binding affinities between the two components and membranes containing phosphatidylcholine (PC) lipids, showing that LukF undergoes the most stable membrane-binding with respect to Hlg2 by means of two distinct binding sites located on one specific side of the rim domain; therefore, the working hypothesis is that LukF is the first component to bind the bilayer in the absence of proteinaceous receptors, and that it mediates the anchoring of Hlg2 by exposing the N-ter interface toward the solvent. The latter is here named interface II, in line with the nomenclature used in reference [10]. These results were used here in setting up the starting configuration of the MD simulations (Figure 1), in which the two proteins are free to diffuse and interact in the absence of any sampling bias. The dynamics reveal that the first contacts between the two proteins are established through the corresponding rim domains, and that this interaction is accompanied by a progressive unfolding of the N-ter of LukF. Modeling of the dimer with protein–protein docking both in the presence and in the absence of the N-ter reveals differences in the best-scoring models, suggesting a possible role for LukF N-ter to guide the inter-molecular interactions that lead to a correct orientation of the two proteins, thus preventing the formation of non-functional dimers. Finally, in the MD simulation of the modeled dimers, we observe peculiar interactions between the two components in the absence of the N-ter, leading to conformational changes in the loop of LukF involved in the interaction with the prestem. This allowed us to make a hypothesis on the possible steps leading to the stem release upon oligomerization.

## 2. Results

### 2.1. Spontaneous LukF-Hlg2 Dimerization on the Membrane Shows a Large Contact Interface through the Rim Domains

Previous works [21] suggested that, in the case of pore formation from γ-HL on simple lipid bilayers and in the absence of proteinaceous receptors, the first interactions between the membrane-bound LukF (LukF_memb_) and Hlg2 in a solution (Hlg2_sol_) take place through interface II—according to the nomenclature used in the description of the crystallographic pore structure [10]—namely the interface exposing the N-ter of LukF. Therefore, to study the process of spontaneous dimerization between LukF_memb_ and Hlg2_sol_, we set-up a system containing both components, where Hlg2 is placed at a distance of about 1.5 nm from interface II of LukF (Figure 1). Three replicas of the LukF_memb_-Hlg2_sol_ system were simulated, with initial velocities independently sampled from a Maxwell–Boltzmann distribution for each run, as explained in Section 4.1. By monitoring the minimum distance between the LukF and Hlg2 components, as shown in Figure S1, it was evident that replica 2 is the only one where a stable interaction between the monomers is established, whereas in the other ones, Hlg2 diffuses away from LukF; in those cases, the simulation was interrupted, while the replica where spontaneous dimerization takes place was extended for 1 μs of the total simulation time. In the following, we report the results from the analysis of this trajectory, by performing comparisons with the simulations of the individual monomers, namely LukF anchored to the membrane (LukF_memb_), and Hlg2 in a solution (Hlg2_sol_).

**Figure 2:**
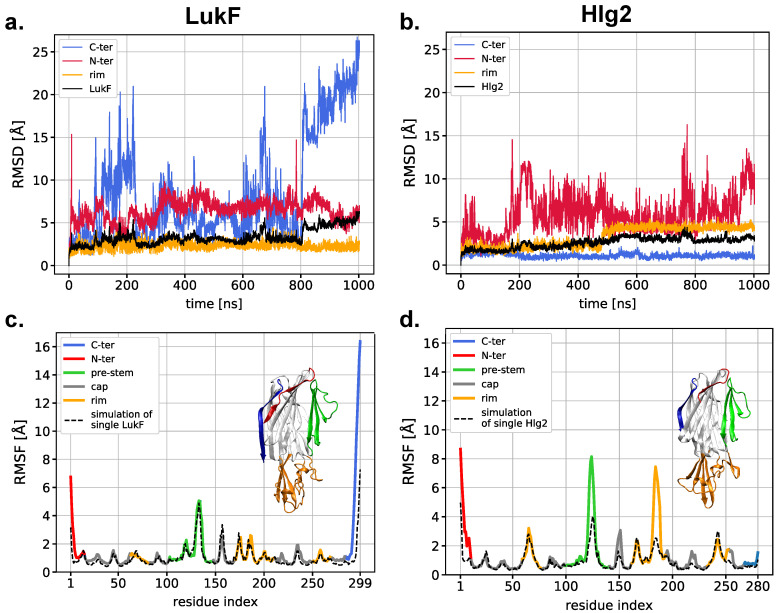
Time evolution of the RMSD for the (**a**) LukF and the (**b**) Hlg2 monomers in the replica where spontaneous dimerization on the membrane is observed. The colored lines correspond to the RMSD computed on different domains of the monomers, while the black line indicates the RMSD of the full proteins. For the same replica, the RMSF of the (**c**) LukF and the (**d**) Hlg2 residues (C${}_{\alpha }$ atoms) (solid lines colored according to the different monomer regions) is reported along with the RMSF of the monomers simulated alone (dashed black line). A graphical representation of the two monomers is also shown for clarity.

A preliminary analysis of the structural mobility of LukF and Hlg2 within our MD simulations shows some noticeable differences between the diverse regions of the two monomers. Figure 2 and Figure S2 report the time evolution of the root-mean-square displacement (RMSD), computed for the $C_{\alpha }$ atoms with respect to the initial frame. In the case of LukF, large deviations are observed in the N- and C-termini, while the rest of the protein is very stable. In Hlg2, on the other hand, large RMSD fluctuations are observed in the N-ter, while the rim abruptly changes its conformation after 500 ns of simulation time. Specifically, this steep change is due to the structural rearrangement of one of the three sequences forming the rim domain, namely the S162-P198 (labeled with letter B in Figure S3, where the Hlg2 structures representative of the two rim conformational states are shown). Figure S2 shows that steep changes along the simulation also affect the LukF and Hlg2 prestems—which contain a particularly mobile loop—especially after ∼400 ns. The comparison between the per-residue values of root-mean-square fluctuations (RMSF) of the two proteins when simulated alone and in the dimerization trajectory (Figure 2) reveals that the fluctuation profiles are strikingly similar, with the exception of the regions highlighted in the discussion above; namely, the N- and C- termini of LukF and the N-terminus, the prestem, and the rim domain (especially the sequence B mentioned above) of Hlg2. This suggests that their observed large fluctuations derive from the interaction between the two proteins, and that they might be involved in the formation of inter-molecular contacts.

The evolution of the contacts between the proteins along the simulation time was monitored by computing the surface area buried at the protein–protein interface—as defined in Section 4.4—distinguishing the individual contributions of the different domains. Figure S4 reveals an increase in the interface area for the first 600 ns of simulation, after which the average value tends to stabilize—especially in the last 200 ns—despite the large fluctuations. Moreover, Figure S4 clearly illustrates that the major contribution to the interface area is attributable to the rim domains in both monomers. In particular, 65% and 95% of the area—averaged over the last 200 ns of simulation—belongs to the rim domain of LukF and Hlg2, respectively. The remaining contact area comes from the cap domain and the C-ter in LukF, and the cap domain, the prestem, and the N-ter in Hlg2. The large contribution of the Hlg2 rim domain to the interface is in agreement with the results from the RMSD and RMSF analyses, which highlight a conformational rearrangement ascribable to the interactions between the monomers.

This result highlights the discrepancy between the interface established in the spontaneous dimerization observed in the simulation and interface II formed by the LukF and Hlg2 protomers in the crystal pore, where the interactions between the cap domains make predominant contributions [10] (excluding the interaction between the stems, which form the β-barrel in the assembled pore), while the rim domains are found in contact with the membrane. A graphical visualization of this difference can be found in Figure S5, where the minimum distance between each LukF residue and the Hlg2 monomer (Figure S5A), and between each Hlg2 residue and the LukF monomer (Figure S5B), averaged over the last 600 ns of simulation, is compared with the same quantity from two protomers in the crystal pore. Similarly, the angle formed between the protein axes (defined as in Section 4.4), converges to a value that is much larger than the one found between adjacent protomers in the assembled pore (Figure S6). However, we stress here that a close resemblance between the dimer and the pair of protomers from the pore structure is not expected, since the large steric hindrance of the folded prestem prevents the cap domain to form the same contacts as in the assembled pore. The two monomers must, therefore, interact through a different, temporary pattern of contacts, which is likely to rearrange upon the formation of the octamer and the unfolding of the prestem.

The analysis of the specific interactions between the two monomers was performed by differentiating between the contribution of H-bonds, salt bridges, and hydrophobic contacts. The residue–residue interaction persistencies between LukF and Hlg2 confirm that the majority of the contacts involve their rim domain, including several H-bonds and a particularly persistent electrostatic contact between K65 in LukF and D181 in Hlg2 (located in the rim sequence undergoing the major structural change, as shown in Figure S3). Persistent H-bonds between the LukF cap domain and Hlg2 rim domain are also established (K29 and Q169, N60, and N167). The LukF C-terminal, particularly terminal residue K299, is also found to form transient salt bridges with some residues belonging to the Hlg2 prestem (K99, D101, D104, and K108). As expected, the simulated dimer showed none of the stabilizing contacts forming the inter-protomers interface II in the crystal pore [10], such as the key salt bridge between D38, located in the loop A (connecting the beta-strands $\beta_{2}$ and $\beta_{3}$, at the top of the cap domain) of Hlg2, and K21 in $\beta_{1}$ of LukF. Indeed, in the LukF monomer the presence of the amino-latch—which is disordered in the protomers of the crystal pore—reduces the accessibility of the adjacent $\beta_{1}$ residues, preventing the formation of the aforementioned contact. The unwinding of the N-ter, which is likely to be triggered by the presence of the Hgl2 monomer and precede the prestem rearrangement and the prepore formation [10,24], is indispensable for the stabilization of the assembled pore. Although a direct interaction between the LukF amino-latch and the Hlg2 monomer has not been observed during the simulation, the former shows an increase in mobility compared to the simulation of the single LukF monomer—as already illustrated by the RMSF in Figure 2. An investigation of the molecular details concerning the amino-latch fluctuations follows in Section 2.2.

### 2.2. Contacts Formed by LukF N-Terminus upon Dimerization

**Figure 3:**
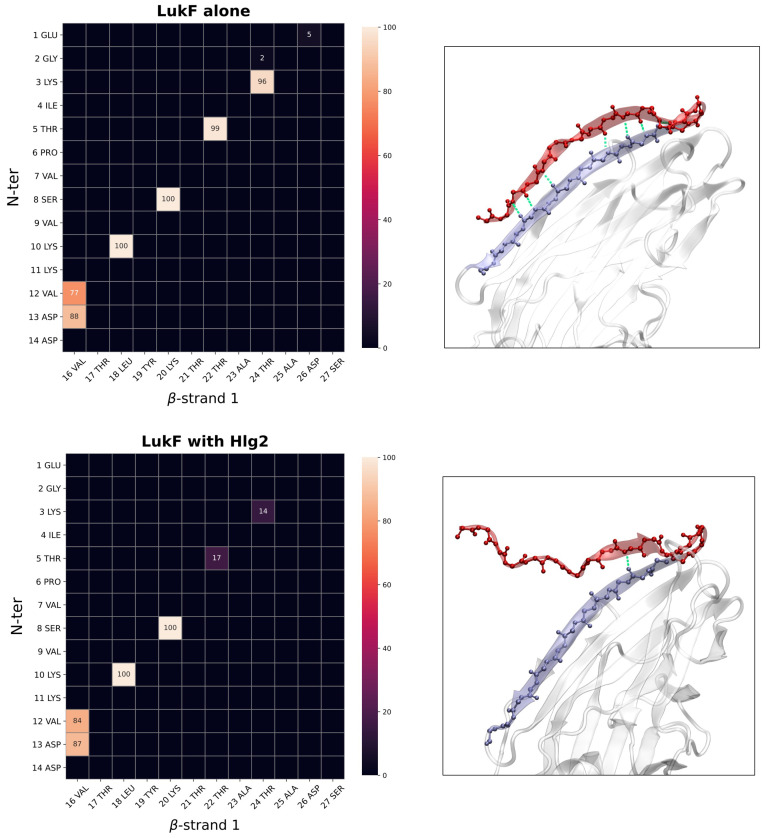
H-bond persistence matrix showing the percentage of simulation frames in which an H-bond contact is formed between the LukF residues (backbone only) of the N-ter and the adjacent beta-strand $\beta_{1}$. The matrices are reported for the simulation of the single LukF (**top**) and for that showing the spontaneous dimerization on the membrane (**bottom**). A graphical representation of LukF showing the N-term (red) H-bonds in the two simulations is also reported.

Starting from the evidence that the amino-latch of the protomers is disordered in the crystal structure of the assembled pore [10] and that its rearrangement is necessary for the formation of pivotal electrostatic contacts at the protomers interfaces (as already discussed in Section 2.1), we investigated the molecular details at the origin of its large fluctuations observed in the simulation of the LukF_memb_-Hlg2_sol_ system. The H-bond persistence matrices between the backbone atoms of the LukF amino-latch and its adjacent beta-sheet ($\beta_{1}$) in the cap domain are reported in Figure 3 and Figure S7 for the LukF_memb_ and the LukF_memb_-Hlg2_sol_ system. A comparison between the two highlights a drastic drop in the persistence of two H-bonds that stabilize the β-sheet, namely the ones formed between K3 and T24 and between T5 and T22. In particular, the disruption or weakening of these bonds results in a partial unfolding of the amino-latch that accounts for the higher mobility of this terminal region. A graphical representation of the LukF N-ter and its adjacent β-strand, reported in Figure 3, clearly illustrates the differences in the amino-latch conformation between the single LukF monomer simulation and the one in presence of Hlg2. The comparative analysis of the intra-LukF interactions involving the N-ter also revealed that, in the LukF_memb_-Hlg2_sol_ system, the first residues of the amino-latch form transient H-bonds (E1-T5) and electrostatic contacts (E1, K3) with some residues in the C-terminal beta-sheet. Specifically, K288, L289, and D291 are involved in the most persistent H-bonds, while K288, D291, and E294 form the most persistent salt bridges. These interactions may have a role in the disruption of the stabilizing H-bonds in the amino-latch. Interesting results also emerge for those hydrophobic contacts reported to couple the amino-latch and the prestem in the crystal structure of the LukF monomer [9], which are indeed found in the simulation of the LukF monomer on the membrane; these include hydrophobic contacts between V12 and A137, and between V16 and A137. The simulation of the LukF_memb_-Hlg2_sol_ system shows a drastic drop in the persistence of these two contacts (Figure S8). Although a transient hydrophobic contact between amino-latch V16 and prestem F118 is established along the simulation of LukF_memb_-Hlg2_sol_, the overall trend appears to be a decrease in the amino-latch/prestem coupling with respect to the simulation of the single LukF.

### 2.3. Protein–Lipid Interactions and Reorientation of the Dimer with Respect to the Membrane Plane

**Figure 4:**
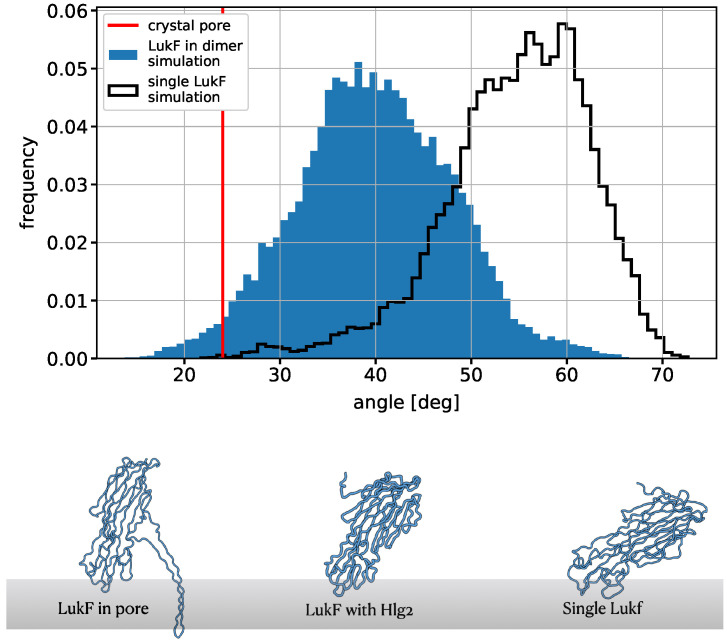
Distribution of the angle between LukF and the axis perpendicular to the membrane. The filled blue histogram refers to the simulation of spontaneous dimerization, while the black empty histogram refers to the simulation of the LukF monomer alone on the membrane. The red dashed line corresponds to the angle formed by the LukF protomer in the crystal structure of the assembled pore (PDB ID: 3B07). The images of the LukF monomer show the differences in LukF orientation between the three stages of pore formation.

The investigation of the contacts between the proteins and the membrane shows a different behavior between the two monomers. As known from previous MD studies [21], LukF is able to form stronger interactions with PC-containing membranes compared to Hlg2 (Figure S9); this is consistent with the stable anchoring of LukF to the bilayer, which persists for the whole duration of the simulation and is mediated by electrostatic interactions between the protein residues and the POPC polar heads, in addition to cation-π interactions between aromatic residues and the choline groups of PC (Figure S10). The binding site identified from the simulation involves residues that are well-known to be responsible for the anchoring of the protein to the membrane [9,21,25], including W176, Y179, H185, T187, Y188, E191, and R197; some of these residues, specifically W176 and R197, have also been shown to steadily bind PC-mimetics in the final pore configuration. However, other residues identified as relevant for the binding of LukF to the membrane, such as Y71, D72, and F73 [21,26], show a much lower tendency to interact with the lipids compared to the case of the single monomer on the membrane. This result, which is the outcome of an evolution of the interaction between LukF and the membrane upon dimerization with Hlg2, is reflected also in the change in orientation of LukF with respect to the axis perpendicular to the bilayer plane (Figure 4). In particular, the distribution of the angle formed between the *z*-axis and the axis along the LukF monomer (defined as the vector connecting the C${}_{\alpha }$ atoms of residues Y252 and L51) displays a substantial difference between the simulation of the single LukF monomer and the one in the presence of Hlg2. In the simulation of LukF_memb_ and at the beginning of the LukF_memb_-Hlg2_solv_ simulation, LukF is bent toward the membrane, exposing interface II, namely the one containing the amino-latch, toward the solvent; this result is expected on the basis of previous experimental and computational works [10,21]. During the course of the dimerization simulation, however, the orientation of LukF changes, and the angle distribution is shifted toward smaller values; in particular, the orientation moves toward the angle characterizing the protomer in the crystal pore (as reported in Figure 4), where the LukF axis is almost perpendicular to the membrane.

No stable lipid-binding site is identified on Hlg2; however, transient contacts with POPC molecules are formed through residues Y66, Y68, and T241 (Figure S11). These interactions are likely responsible for the reorientation of the dimer with respect to the membrane plane and for the consequently larger exposure of interface I of LukF to the solvent. This change of orientation might, therefore, be functional for the interaction with a second membrane-anchored dimer through interface I.

### 2.4. The Presence of LukF N-Terminus Impacts the Modeling of the Dimer with HADDOCK

The above-mentioned MD simulations of spontaneous LukF-Hlg2 dimerization, performed in absence of any bias, provide an insight into the first steps of the interaction between the two proteins. However, sampling problems in unbiased MD—related to the timescale of the dimerization process—do not allow capturing the formation of the final dimer, where all of the stabilizing intermolecular contacts are established. To circumvent this problem, we combined MD simulations with protein–protein docking through the HADDOCK2.4 web server [27,28]. HADDOCK employs an information-driven semi-flexible docking approach, starting from an equilibrated configuration of the single LukF and Hlg2 monomers (generated by means of the simulations of the LukF_memb_ and the Hlg2_sol_ systems, respectively). The docking was driven by the information coming from the inter-protomer contacts of interface II in the crystal structure of the pore [10], namely the same interface studied in the above-mentioned MD simulations. Given the partial unfolding of LukF amino-latch observed in the simulation upon interaction with Hlg2, and the hypothesis of the necessity of its full unfolding for the creation of a stable dimerization interface, we decided to investigate its role in the formation of a stable dimer by modeling the LukF-Hlg2 complex both in the presence and the absence of the first 15 residues of the N-terminal region of LukF; the length of this segment was chosen according to the part of the protein that results unfolded in the structure of the pore [10]. In this setup, the removal of the amino-latch is intended to mimic its full unfolding and, therefore, the exposure of the $\beta_{1}$ strand of LukF. Additional details about the docking procedure, the parameters and the restraints adopted are reported in Section 4.2.

**Figure 5:**
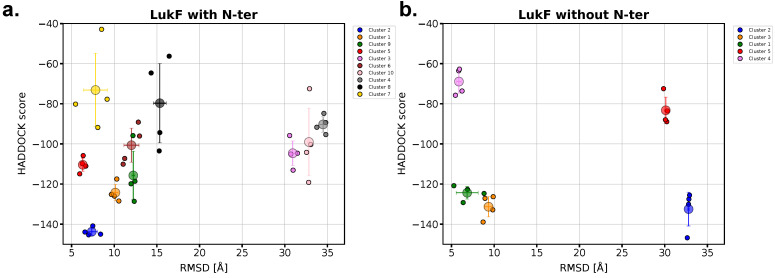
Plots of the HADDOCK scores of the predicted LukF-Hlg2 dimers (in the presence (**a**) and absence (**b**) of the LukF amino-latch) and their RMSD computed with respect to the same dimer extracted from the crystal structure of the pore. Each color represents a specific cluster of structures generated by HADDOCK. For each cluster, the data referring to the four top-scored structures (small circles) are shown, along with their average values and standard deviations (larger circles with bars).

The HADDOCK protocol generates and refines several models of the protein complex, ordered according to an energy-based scoring function (as defined in Section 4.2) and clustered by means of a similarity measure, namely the fraction of common inter-molecular contacts. The centroids of each cluster were further analyzed to identify and compare the best-scoring models. First, a structural comparison between every single monomer (the starting structure used by HADDOCK to generate the models) and the same monomer in the HADDOCK dimer revealed the absence of local structural rearrangements (RMSD value < 1 Åand single residue displacements < 2 Å). In addition, differences in the relative arrangements of the two proteins in each model with respect to the LukF-Hlg2 complex in the crystal pore were evaluated by computing the RMSD on the C${}_{\alpha }$ atoms of Hlg2, after an alignment performed over the C${}_{\alpha }$ atoms of LukF. Given the large differences in their conformation before and after pore formation, the residues belonging to the prestem/stem domain and the N-ter were excluded from the analysis. The results are reported in Figure 5 for the four top-scored models of each cluster, both in the presence and absence of the LukF N-ter. We notice that the number of clusters generated by HADDOCK is 10 in the presence of the LukF amino-latch and only 5 when the N-ter is absent, which indicates fewer possible binding modes for the N-ter truncated complex. In the former case, the best-scored cluster (cluster 2) is also the one containing the lowest RMSD values. There appears also an evident gap in the score that separates the models belonging to cluster 2 from the other ones; this leads to the straightforward choice of the top scorer for cluster 2. In the absence of the LukF N-ter, instead, the clusters containing the best scored models share a similar score, and the overall top-scorer (belonging to cluster 2) corresponds to a very large RMSD value. A visual inspection reveals that this predicted complex displays a “non-functional” arrangement between the monomers, having LukF and Hlg2 a reciprocal orientation∼$180^{\circ }$ flipped with respect to the adjacent protomers in the crystal pore (Figure S12). For this reason, the second top-scored model, corresponding to cluster 3, was chosen as a representative structure of the dimer in absence of the amino-latch.

**Figure 6:**
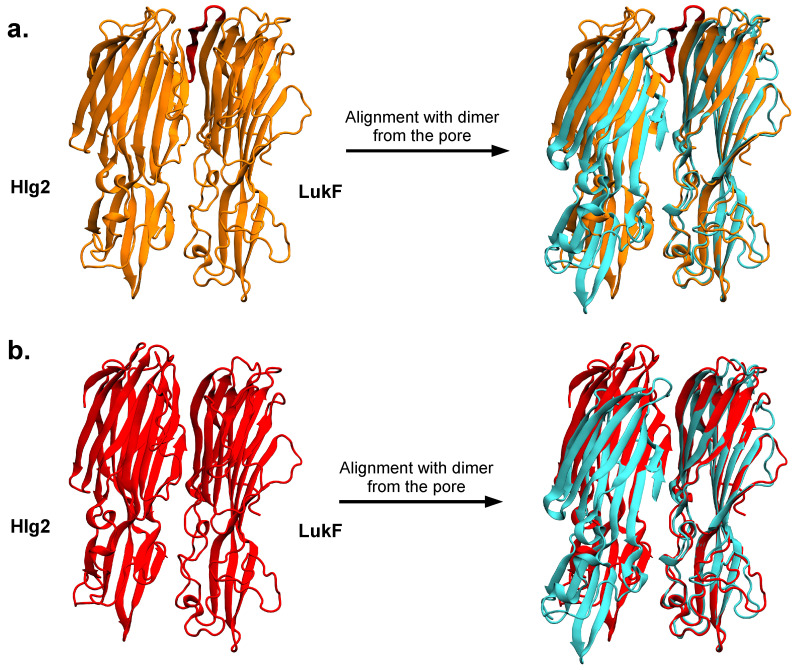
Graphical representations of the HADDOCK models of the Hlg2-LukF dimer in the presence (**a**) or in the absence (**b**) of the LukF amino-latch. The comparison with the two adjacent protomers in the final pore (colored in cyan) is performed after structural alignment on LukF alone. In the comparison with the pore dimer, the stem/prestem domains are not shown for clarity.

A graphical representation of the selected HADDOCK models, both in the presence and in the absence of the N-ter, confirms that the relative arrangement of the two components is very similar (Figure 6). The structural alignment between the representative models of the dimer and the two adjacent protomers in the crystal pore (Figure 6) reveals that the largest deviations between the models and the crystal structure mainly concern the rim domains and the top of the cap domains, which are involved in stabilizing contacts between nearby protomers of the octamer. This is also highlighted in the plots depicting the displacements of the Hlg2 C${}_{\alpha }$ atoms between the HADDOCK dimer and the dimer in the crystal pore, after a structural alignment performed on the LukF monomers (Figure S13). We remark that, even in the absence of the N-ter, the modeled dimers lack the pivotal electrostatic interaction that is established in the pore interface II between D38, located in the loop A of the Hlg2 cap domain, and K21 in the LukF $\beta_{1}$ strand. Figure 6 reveals that the formation of this contact in the modeled dimers is not possible due to the presence of the folded prestem of Hlg2, which prevents the Hlg2 loop A to come in contact with the LukF $\beta_{1}$ strand. This is in line with the fact that further conformational changes, involving a complex interplay between the formation of intermolecular interactions and the prestem release, are expected to take place after the dimer is formed. For this reason, the temporal evolution of the contacts between the two proteins, starting from the docking models, was further studied by means of MD simulations, as described in the next section.

### 2.5. Simulations of the Modeled Dimers Reveal Conformational Changes of LukF in Absence of the N-Terminal

Simulations of the chosen representative structures of the modeled dimers—obtained in the presence and absence of LukF N-ter—were performed in a solution, collecting two 500 ns-long replicas for each system. In this setup, the absence of the membrane, which allows the protein complex to equilibrate faster, is justified by the experimental observation of the presence of dimers in a solution [29].

**Figure 7:**
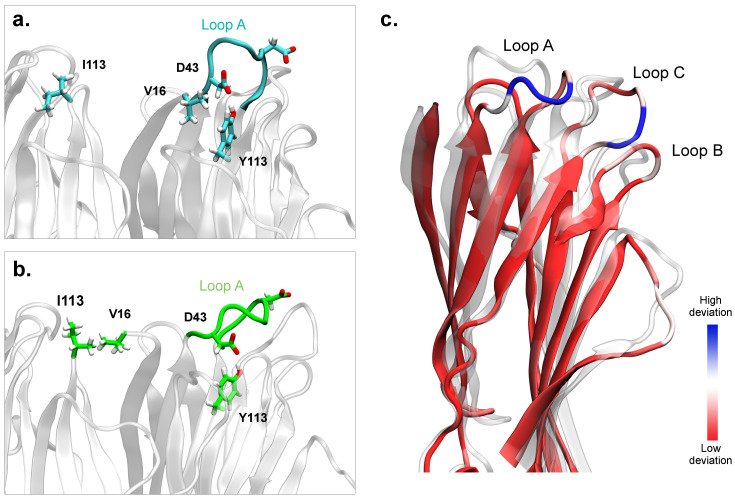
Representations of the cap domain of LukF in the dimer obtained from HADDOCK without the N-ter, at the beginning (**a**) and at the end (**b**) of the 500 ns-long MD simulation. (**c**) Structural alignment of the starting and final structures of the cap domain of LukF. The former is represented as a semi-transparent cartoon, while the latter is colored according to the RMSD between the two conformations. The largest deviations are observed in loop A (residues 43–48) and loop C (residues 232–237).

The protein–protein interface remains stable in the presence of the N-ter of LukF. On the contrary, in one of the replicas performed in the absence of N-ter, the residue V16 of LukF undergoes a dramatic transition, reorienting itself toward Hlg2 (Figure 7 and Figure S14). In the initial models, and in the other simulations performed, V16 is engaged in hydrophobic interactions with A137 and Y113 of LukF prestem; following the transition, it forms a hydrophobic contact with I113 located on the prestem domain of the adjacent Hlg2 monomer (Figure 7). This behavior is not observed in the simulations that include the LukF N-ter. Given the amplitude of this conformational change, it is indeed very unlikely that the backbone of V16 can undergo such a large motion when conformationally restricted by the folded amino-latch; moreover, the presence of the beta-strand of the N-ter creates a steric hindrance that separates V16 from the Hlg2 surface. On the other hand, a complete unfolding of the N-ter might confer sufficient backbone plasticity to enable such a transition, and it would remove any steric hindrance between the two protein surfaces.

The sudden reorientation of V16 is accompanied by the transient breaking of the H-bond between the same Y113 residue of the prestem and D43, located on loop A; the latter is the loop connecting β2 and β3 of LukF, and includes residues from A43 to K48 (Figure 7). The breaking of this interaction leads to a rearrangement of loop A and the neighboring ones, and the subsequent formation of the H-bond between the same two residues after the loop reaches its new conformation. In this new configuration, the loops located in the upper region of the cap domain (in particular, loop A and loop C) are oriented far from the interface with Hlg2 (interface II), and extend toward the opposite side of the protein (interface I). The relevance of this conformational change lies in the fact that a structural perturbation of these loops is also observed in the structure of the final pore, where loop A is engaged in interprotomer electrostatic interactions with the β1 strand of the protomer in contact through interface I; therefore, it might be functional for the interaction with a second dimer during the oligomerization stage.

The H-bond between A43 and K48 is considered a key interaction stabilizing the folded state of the prestem in the monomeric form of LukF [9,10], because it anchors the prestem to the cap domain. The breaking of this interaction, the release of the prestem, and its unfolding are all necessary steps for the subsequent formation of the transmembrane β-barrel, where the stem of each protomer refolds as two long β-strands. Although the process leading to the prestem release is not known, it is supposed to take place only after a multimeric assembly is formed. Therefore, it is likely that several sequential steps are required, first involving the reorientation of the loops located at the top of the cap domain of the single protomer and, subsequently, their interactions with adjacent dimers.

## 3. Discussion and Conclusions

In this work, we investigated the dimerization process of LukF and Hlgs, the two components of the *S. aureus* leukotoxin γ-HL. Unbiased simulations of spontaneous dimerization highlight the peculiar behavior of the N-terminal domain of LukF, which tends to unfold upon interaction with Hlg2. This behavior is in line with the available experimental data: in the crystallographic structure of LukF in the monomeric form, the N-ter is folded as a short β-strand together with the cap domain [9], while in the prepore [8] and pore experimental [10] structures, the LukF amino-latch is not resolved, suggesting that it is disordered. A structural alignment between the monomer and protomer structures would lead indeed to an overlap between the N-ter of LukF and the cap of the nearby Hlg2 protomer. On the other hand, an experimental structure of the monomeric Hlg2 component is not available; however, in the structure of the covalent Hlg2-LukF heterodimer, where the two monomers interact through the interface of Hlg2 exposing the N-ter and the one of LukF opposite to the N-ter, the amino-latch of Hlg2 is not resolved and, therefore, unfolded [12]. Taken together, these observations suggest that the N-terminal regions at the interface between components should be released from the cap domain before the inter-molecular contacts (which are needed for the formation of a functional dimer) are established. In our simulation of spontaneous dimerization between membrane-bound LukF and Hlg2 in a solution, the partial unfolding of the amino-latch of LukF upon interaction with Hlg2 takes place after the first contacts between the rim domains of the two proteins are established. These interactions are accompanied by a progressive increase in the contact surface area of interface II between the proteins, and by the reorientation of the dimer with respect to the membrane plane. Specifically, the angle between the dimer axis and the normal to the membrane plane is reduced by approximately 20 degrees, thus leading to a larger solvent exposure of interface I of LukF, which was almost parallel to the bilayer in the initial conformation. Such behavior might be necessary for the subsequent oligomerization through interface I with another membrane-bound dimer. In this step, a role might be played by the observed increase in the flexibility of the C-ter of LukF; however, the formation of larger oligomers will be the topic of future studies.

To date, little is known about the functional role of the N-terminal regions of bi-component leukotoxins, whose sequences are highly conserved among toxin components [30]. Our results from modeling the LukF-Hlg2 dimer, both in the presence and absence of the LukF amino-latch, show that a non-functional dimer model, in which the two monomers have opposite orientations, is energetically more plausible than the functional dimer resembling the pore subunit when the amino-latch is absent. However, the formation of such a non-functional dimer is much less favored when the N-terminal regions are preserved. This allowed us to make a hypothesis for a possible role of the N-terminal in guiding the interactions between the two components: specifically, the presence of the folded amino-latch in the monomeric form of the protein might prevent the formation of non-functional dimers, which become energetically more favorable in the absence of the N-ter. Published experimental observations showed that the cytotoxic activities of γ-HL toward erythrocytes are not affected by the truncation of the N-terminal region of LukF [31] (similar to what happens with the amino-latch region of Hlg2, which is dispensable for prestem release in the formation of transmembrane β-barrel pores [30]). On the contrary, the deletion, including the three subsequent residues at the N-ter of LukF, impaired the formation of pores because of the largely reduced binding activity on erythrocyte surfaces, compared to that of intact LukF. These results suggest that the N-ter region of LukF might be involved in the interaction between LukF and proteinaceous receptors on the membrane of the target cell, which was recently identified as the atypical chemokine receptor 1 ACKR1, although a structural model of the complex is still missing [29,32]. In this case, the formation of a non-functional LukF-Hlg2 dimer in the absence of LukF N-ter is not possible since the same LukF interface is already involved in interactions with the cell surface and the interaction with Hlg2 has to take place through the opposite face of LukF (interface I). In addition, in such an experimental setup, the formation of the non-functional dimer could not take place in a solution since the protocol used to measure the cytotoxic activity followed a two-step procedure, where the Hlg2 component was added to the reaction mixture only after incubation of the erythrocytes with LukF [31,33]. In the case of pore-formation on simple lipid bilayers, in the absence of additional proteins, the binding mechanism of LukF was suggested to differ [21,22]: in this case, the N-ter does not interact directly with the lipids, because the anchoring of the protein to the membrane is mediated by two phosphatidylcholine binding sites located on the rim domain [9,21,25,26]. Therefore, the hypothesis of the role of N-ter of LukF as a guide for the correct relative orientation of the two monomers is not in contradiction with the available experimental data; additional experiments on model membranes are needed to verify whether the N-ter of LukF can facilitate the proper orientation of Hlg2 for the formation of a functional dimer.

For the closely-related monocomponent α-hemolysin, it has been shown that the amino-latch works as a molecular switch for the release of the prestem [9,34]; conversely, it has been hypothesized that γ-HL possesses an amino-latch-independent prestem release mechanism, a role that might be instead played by specific interactions at the interface between protomers [35,36]. In the case of the Hlg2-LukF interaction through interface I (therefore, the opposite interface with respect to the one considered in our study), it was suggested that the electrostatic interaction between R16 of Hlg2 and D122 on the prestem of the adjacent LukF might serve as a switch for the stem release of Hlg2 from the cap [36]. However, unlike Hlg2, the corresponding basic residue of LukF on interface II, namely K21, was not suggested to work as a molecular switch, since an acidic residue is absent in the prestem of Hlg2 [30]. Our MD simulations of the functional models of the LukF-Hlg2 dimer obtained by HADDOCK reveal conformational changes in the upper region of the cap domain of LukF in the absence of the N-ter, namely the interaction between residue V16 on the cap of LukF and I113 on the prestem of Hlg2, accompanied by the reorientation of loops A and C, which anchor the prestem to the cap domain through H-bonds. The hydrophobic interaction between V16 of LukF and I113 of Hlg2 in interface II might, therefore, take the role of the salt bridge observed in interface I. However, additional experimental studies are necessary to better understand how the intermolecular contacts between the toxin components lead to the stem release, and the formation of the transmembrane β-barrel.

## 4. Materials and Methods

### 4.1. System Setup

The LukF and Hlg2 structures employed for the MD simulations and the dimer modeling via protein–protein docking were obtained from the Protein Data Bank (PDB) crystals of the LukF monomer (PDB ID: 1LKF [9]) and of the soluble LukF-Hlg2 covalent dimer (PDB ID: 2QK7 [12]), respectively. The LukF monomer crystal displayed a disordered region between residues 130–136, which was reconstructed using the software MODELLER [37], as done in [21]. The Hlg2 structure, instead, was isolated from the engineered hetero-dimer crystal and the cysteine residue (C28), involved in the disulfide bridge with the LukF counterpart, was mutated back to the wild type threonine residue, as done in [21]. This Hlg2 structure also lacked the disordered 10 residue long N-terminal domain, which was modeled with AlphaFold [38] via the ChimeraX AlphaFold tool [39].

The resulting structures were employed to perform unbiased atomistic MD simulations of the two components separately, namely (i) the LukF monomer alone on the membrane (LukF_memb_) and (ii) the Hlg2 monomer alone in a solution (Hlg2_sol_), which served as a control for the comparative analysis with the subsequently simulated systems. In order to study the spontaneous dimerization of the two components on the membrane, we then carried out a series of unbiased atomistic MD simulations, starting from the LukF monomer anchored to the membrane—obtained from an equilibrated conformation of the simulated LukF_memb_ system—and the Hlg2 monomer placed close in a solution (LukF_memb_-Hlg2_sol_). The systems containing the membrane were all set up with the CHARMM-GUI interface [40], while the Hlg2_sol_ system was built with the GROMACS 2020 software [41]. All the simulations were performed using the force field CHARMM36m [42,43], with TIP3P model water molecules and K^+^ and Cl^−^ ions to neutralize the system, and to reach a 150 mM ion concentration. In the Hlg2_sol_ system, the Hlg2 monomer was put in a solution in a simulation box of size 11 × 11 × 11 nm^3^. In the LukF_memb_ system, the LukF monomer was placed in a solution with its rim domain in contact with the membrane surface at a distance smaller than the cut-off used for the non-bonded interactions (1.2 nm)—in order to enhance the probability of obtaining a stable anchoring—in a simulation box of size 10 × 10 × 14 nm^3^. The model membrane was built as a flat bilayer spanning the *xy*-plane of 1-palmitoyl-2-oleoyl-sn-glycero-3-phosphocholine (POPC) and cholesterol (in molar ratio 3:1), a composition experimentally shown to allow for the gamma-hemolysin pore formation [22]; moreover, recent results from MD simulations revealed the ability of the LukF monomer to form a stable binding with this kind of membranes [21]. For the setup of the LukF_memb_-Hlg2_sol_ system, we took an equilibrated conformation of LukF coming from the simulation of the LukF_memb_ system and we placed it on a POPC and cholesterol bilayer membrane (in molar ratio 3:1) in a box of an approximate size of 13 × 13 × 15 nm^3^. In order to test the hypothesis that the membrane-bound LukF promotes the Hlg2 anchoring by the presence of a solvent-exposed interface, we placed the Hlg2 monomer in a solution close to the LukF-exposed interface (containing the amino-latch), as shown in Figure 1. The initial distance between the two proteins was larger than the cut-off for the non-bonded interactions.

### 4.2. Protein–Protein Docking

The modeling of the LukF-Hlg2 complex through protein–protein docking was performed using the HADDOCK2.4 web server [27,28], which allows integrating experimental data as restraints and using these to guide the docking process alongside traditional energetics and shape complementarity. The information concerning the interface shared by the LukF and Hlg2 protomers in the crystal structure of the pore was employed by defining distance restraints (called ambiguous interaction restraints, AIRs) that were incorporated in the energy function used for the calculation. The AIRs were defined, for each monomer, through a list of residues (active residues), which were restrained to be a part of the interface, if possible, otherwise incurring a scoring penalty. A residue of a monomer was considered active if its minimum distance from the other monomer in the crystal structure of the pore (through interface II) [10] was smaller than 6.5 Å. The residues belonging to the stem-domain—the region undergoing the conformational change that leads to the beta-barrel formation—were excluded, along with those in the N-terminal region, disordered in the crystal pore. The list of the selected active residues of LukF and Hlg2 is reported in Section S1 of the Supplementary Information. To perform the docking, we employed the structure of the LukF and the Hlg2 monomers resulting from the equilibration phase of the LukF_memb_ and the Hlg2_sol_ system, respectively. The dimer models were generated and refined following the standard docking protocol of HADDOCK, which consists of three different stages, namely (i) randomization of orientations and rigid-body minimization, (ii) semi-flexible simulated annealing in torsion angle space, and (iii) refinement in the Cartesian space with an explicit solvent, keeping the default setting. The detailed information regarding the docking procedure and the default setting parameters can be found at the HADDOCK2.4 site (https://wenmr.science.uu.nl/haddock2.4/ (accessed on 8 November 2022)). The standard HADDOCK protocol generated 1000 models in the rigid body minimization stage, and then refined the best 200—regarding the energy function—in stages (ii) and (iii). The final models were then ranked on the basis of an HADDOCK scoring function, which consists of a linear combination of various energies and is defined as follows: HADDOCK_score_ = $1.0E_{vdw}+0.2E_{elec}+1.0E_{desol}+0.1E_{air}$, where the terms in the sum refer to van der Waals, electrostatic, desolvation, and distance restraint energy, respectively. The final models were then automatically clustered by means of a similarity measure, namely the fraction of common intermolecular contacts.

### 4.3. Simulation Details

All of the simulation runs were performed with GROMACS 2020 software [41] in the NPT ensemble. The temperature was controlled at 310 K with the Nosé–Hoover thermostat [44,45] with a coupling constant of $\tau_{T}=1$ ps. The pressure was set to 1 bar by means of the Parrinello–Rahman barostat [46] with a coupling constant of $\tau_{T}=5$ ps. In the systems containing the membrane, the *xy*-plane and the *z*-axis were independently coupled to the pressure bath. Particle mesh Ewald was employed to treat the electrostatic interaction and a cut-off of 1.2 nm was used for the non-bonded interactions. LINCS algorithm was used to constrain the bonds containing hydrogen atoms. A time step of 2 fs was adopted, along with periodic boundary conditions in all of the spatial directions. The initial velocities were sampled from a Maxwell–Boltzmann distribution. One replica of the Hlg2_sol_ system was simulated for 300 ns after an energy minimization and a two-step 0.5 ns equilibration phase (NVT and NPT). The systems containing the membrane underwent the same energy minimization but a longer six-step equilibration phase (NVT and NPT), for a total of 1.875 ns, where the position restraints were progressively released. One replica of the LukF_memb_ system was run for 700 ns, while three replica of the LukF_memb_-Hlg2_sol_ were simulated. Two initial replica were run starting from the configuration shown in Figure 1 with initial velocities independently drawn from a Maxwell–Boltzmann distribution. In replica 1, since the Hlg2 monomer started diffusing in the solvent after 10 ns, the simulation was stopped after 70 ns. The other replicas were instead launched, starting from the configuration obtained after 200 ns of simulation of replica 2, always adopting random initial velocities. Since the Hlg2 monomer eventually diffused in this simulation, the simulation of replica 2 was the only one that was carried out for 1 μs.

Simulations of the best HADDOCK models, both in the presence and in the absence of LukF N-ter, were performed using the same protocol as in the case of the individual monomers, after building the simulation box with CHARMM-GUI. The dimers were simulated in a 150 mM KCl solution, without the presence of the membrane. Two 500 ns-long replicas were collected for each of the two systems, for a total of 2 μs of simulation time.

### 4.4. Analysis

The analyses of the MD simulations were performed via GROMACS utilities [41] (gmx mindist, gmx gangle, gmx sasa), as well as in-house Python and TCL scripts. The interface area between the two monomers in the simulation of the LukF_memb_-Hlg2_sol_ system was defined through the solvent-accessible surface area (SASA) of the single LukF monomer (SASA_LukF_), of the single Hlg2 monomer (SASA_Hlg2_), and the complex, including both LukF and Hlg2 (SASA_LukF-Hlg2_), as follows:

$$A_{\mathrm{interface}}=({SASA}_{\mathrm{LukF}}+{SASA}_{\mathrm{Hlg}2}-{SASA}_{\mathrm{LukF}-\mathrm{Hlg}2})/2$$

The analysis of the non-bonded interactions was carried out with an in-house Python 3 compatible version of the PyInteraph software [47] (available on request), which we employed to detect the presence of H-bonds, electrostatic contacts (or salt bridges), and hydrophobic contacts, defined by means of geometrical restraints. According to the PyInteraph default criteria, an H-bond is identified when the distance between the acceptor atom and the hydrogen atom is lower than 3.5 Å, and the angle formed between the donor, the hydrogen, and the acceptor is greater than 120°. Where not explicitly specified, the H-bonds we report in the paper can include both the side chain and the backbone atoms. A salt bridge is formed between two different residues if at least one pair of atoms belonging to the charged groups of the two residues is found within a distance of 4.5 Å. In Asp and Glu residues, the atoms forming the carboxylic group are considered, while for Lys, Arg, and His (if protonated) residues, the NH_3_^+^, the guanidinium group, and the imidazole ring are employed, respectively. A hydrophobic contact is defined when the centers of mass of the side chains of two hydrophobic residues are found at distances shorter than 5 Å. The residues considered for hydrophobic interactions are Ala, Ile, Val, Leu, Phe, Met, Trp, and Pro.

The angle between the proteins was measured with the gmx gangle tool of GROMACS, after defining the protein axes as the axes passing through the C${}_{\alpha }$ atoms of residues D44 and W74 of Hlg2 and through the C${}_{\alpha }$ atoms of residues Y252 and L51 of LukF. The analysis of the interactions between the proteins and the POPC lipids of the membrane was performed with the PyLipID tool [48], using a cutoff of 0.6 nm. Graphical representations of the proteins have been produced with VMD [49].

## Data Availability

The raw data associated with this work are freely available on a Zenodo repository at the following link: https://zenodo.org/record/7805866#.ZC7buexBw-Q.

## Supplementary Materials

The following supporting information can be downloaded at: https://www.mdpi.com/article/10.3390/ijms24087113/s1.
