# Supplementary material for: Gamma-Hemolysin Components: Computational Strategies for LukF-Hlg2 Dimer Reconstruction on a Model Membrane
Source: Int J Mol Sci. 2023 Apr 12;24(8):7113. doi: 10.3390/ijms24087113 (PMC10138441; doi:10.3390/ijms24087113)
Supplement: Supplementary file 1 [file ijms-24-07113-s001.zip › ijms-2327587-supplementary.pdf]

Supporting Information

Gamma-Hemolysin Components: Computational Strategies for LukF-Hlg2 Dimer Reconstruction on a Model Membrane

Costanza Paternoster[1,2], Thomas Tarenzi[1,2], Raffaello Potestio[1,2], Gianluca Lattanzi[1,2,*]

[1]*Department of Physics, University of Trento, via Sommarive 14, I-38123 Trento, Italy*

[2]*INFN-TIFPA, Trento Institute for Fundamental Physics and Applications, via Sommarive 14, I-38123 Trento, Italy*

[*] Email address: gianluca.lattanzi@unitn.it

S1 Protein-protein docking with HADDOCK2.4

List of the index of the active residues incorporated in the Ambiguous Interface Restraints, selected as reported in Section 2.2 of the main text:

- LukF: 18, 19, 20, 21, 22, 23, 24, 25, 26, 27, 32, 33, 34, 35, 36, 37, 38, 39, 41, 55, 57, 58, 59, 60, 61, 210, 212, 213, 215, 216, 219, 220, 221, 222, 223, 224, 285, 286, 287, 288
- Hlg2: 38, 39, 40, 41, 42, 43, 89, 90, 91, 92, 93, 94, 96 , 97, 143, 144, 145, 146, 147, 148, 149, 150, 151, 152, 155, 159, 161, 171, 173, 214, 216

S2 Supplementary figures

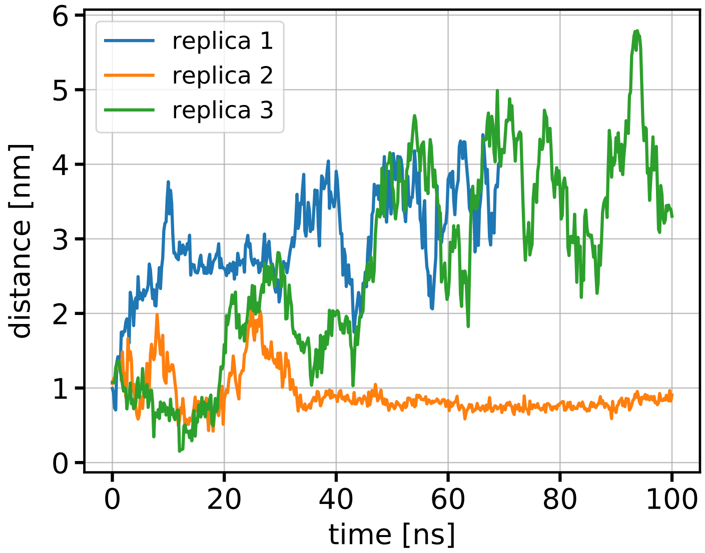

Figure S1: Minimum distance between the LukF and the Hlg2 monomers as a function of time for the three simulated replica of the $LukF_{memb}$-$Hlg2_{sol}$ system. For each monomer we considered only those residues which are found in interface II in the crystal structure of the pore within a distance of 6.5 Å with respect to the adjacent protomer. We excluded the stem-domain and instead included the LukF amino-latch residues.

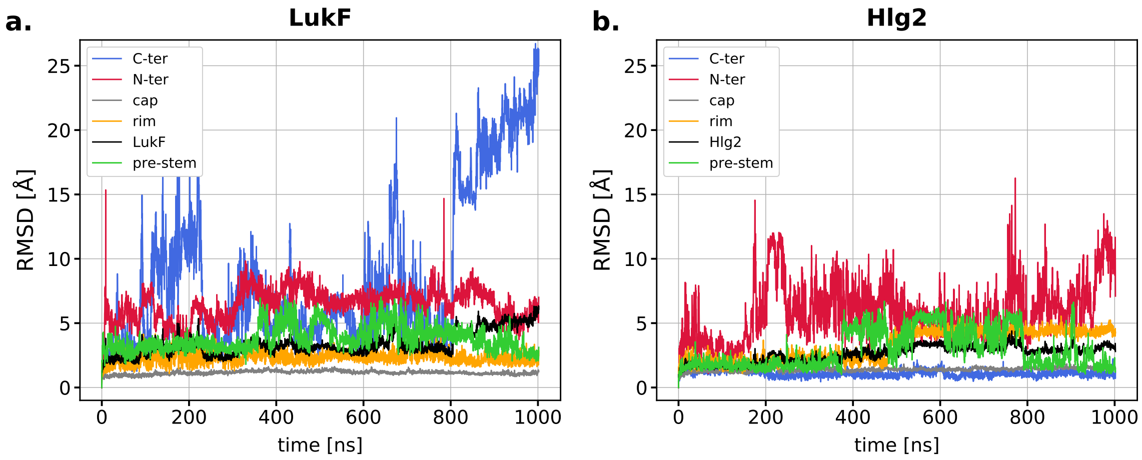

Figure S2: RMSD, as a function of time, plotted for the (**a**) LukF and the (**b**) Hlg2 monomers in the replica where the spontaneous dimerization on the membrane is observed. The coloured lines correspond to the RMSD computed on different domains of the monomers, while the black line indicates the RMSD of the full proteins.

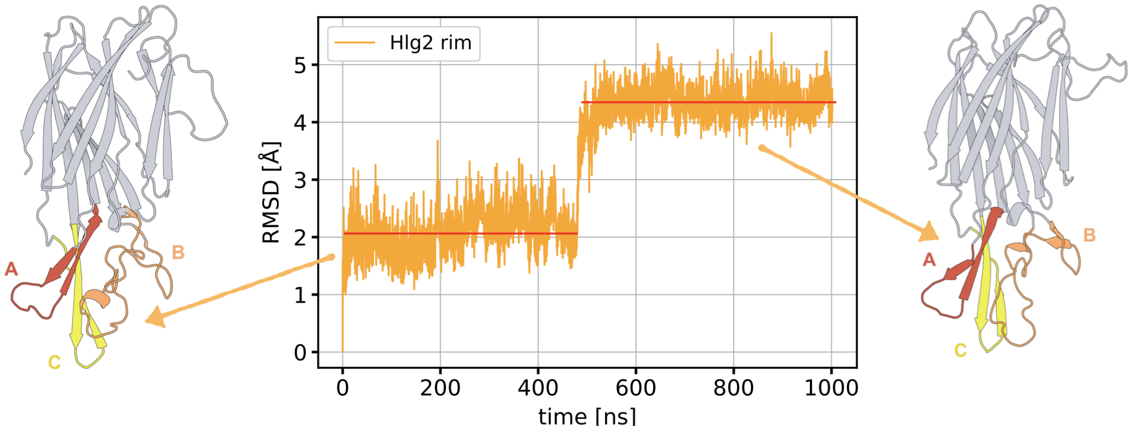

Figure S3: RMSD, as a function of time, plotted for the Hlg2 rim domain in the simulation capturing the spontaneous dimerization. The average values computed before and after the abrupt change at $\sim$ 500 ns are reported (red lines). The images of two Hlg2 structures representative for these two RMSD states are shown, with the three rim domain sequences (A: 59-76, B: 162-193, C: 233-252) coloured for clarity. It is to notice that the sequence B is the one undergoing the major structural change.

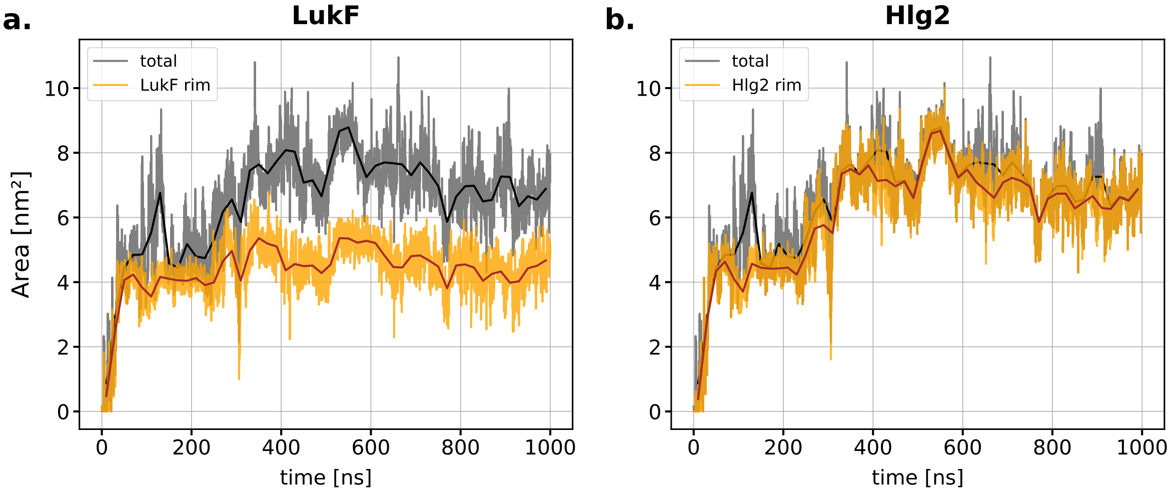

Figure S4: Interface area between the LukF and the Hlg2 monomers in the replica where the spontaneous dimerization on the membrane is observed, as a function of time. The gray line in (**a**) and (**b**) represents the total interface, while the yellow line represents the contribution of the rim-domain of (**a**) LukF and of (**b**) Hlg2 to the interface. A 20 ns average is also reported (black and brown lines).

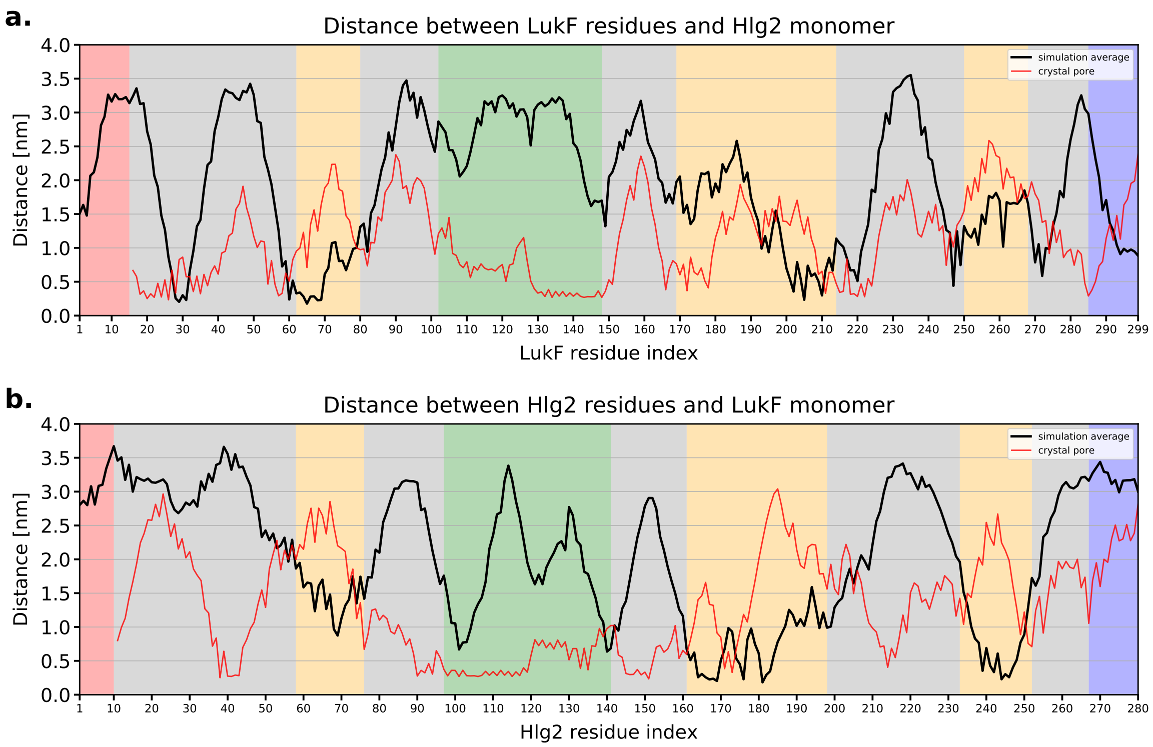

Figure S5: Minimum distance between (**a**) each LukF residue and the entire Hlg2 monomer and between (**b**) each Hlg2 residue and the entire LukF monomer, as a function of (**a**) LukF residue index and (**b**) Hlg2 residue index. The black line represents the average value over the last 600 ns of simulation in the replica where the spontaneous dimerization on the membrane is observed. The red line represents the average value found for the LukF-Hlg2 pairs sharing the interface II in the crystal structure of the pore (PDB ID: 3B07). The coloured areas indicate the different domains of the Hlg2 monomer: cap (gray), pre-stem (green), rim (orange), C-ter (blue), N-ter (red).

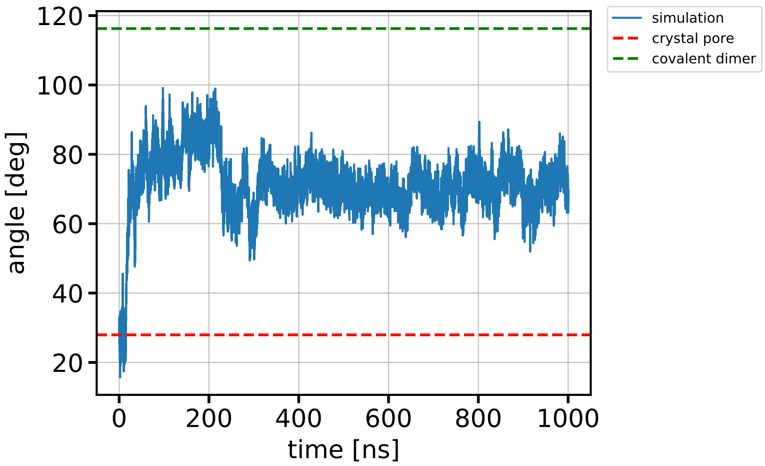

Figure S6: Angle between the LukF axis and the Hlg2 axis – as defined in section 2.4 – as a function of the simulation time in the replica where the spontaneous dimerization on the membrane is observed (blue line). The dashed lines represent the same angle between the protomers in the crystal structure of the pore (red) and between the monomers in the covalent hetero-dimer (green).

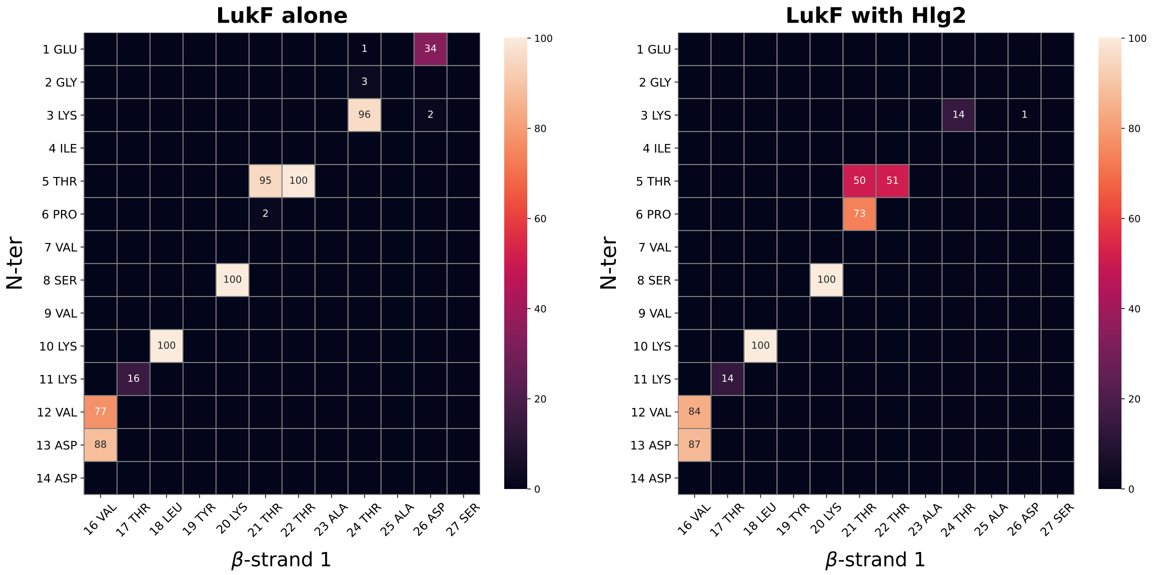

Figure S7: H-bond persistency matrix showing the percentage of simulation frames in which an h-bond contact is formed between the LukF residues of the N-terminal and the adjacent beta-strand β_1, taking account for both the backbone and the side-chain contribution. The matrices are reported for the simulation of the single LukF (left) and for that showing the spontaneous dimerization on the membrane (right).

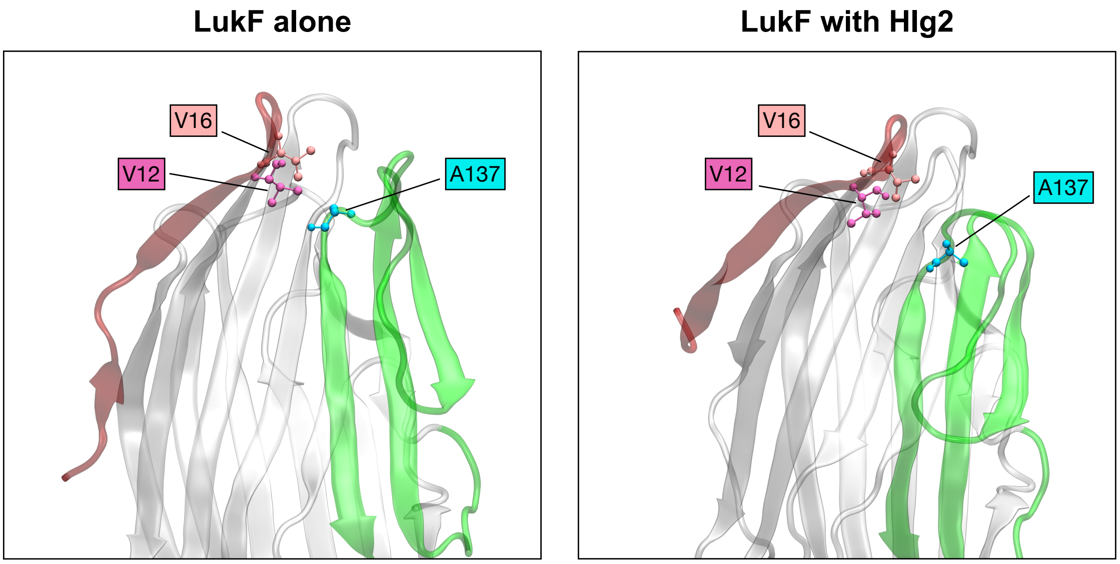

Figure S8: Graphical representation of the hydrophobic contacts formed in the LukF monomer between V12 and A137 and between V16 and A137 – reported to couple the N-ter (red) and the pre-stem (green) in the crystal structure of the LukF monomer – in a frame of the single LukF monomer simulation (left). The same residues are also shown for a frame extracted from the simulation capturing the spontaneous dimerization on the membrane (right), where the abovementioned hydrophobic contacts are disrupted.

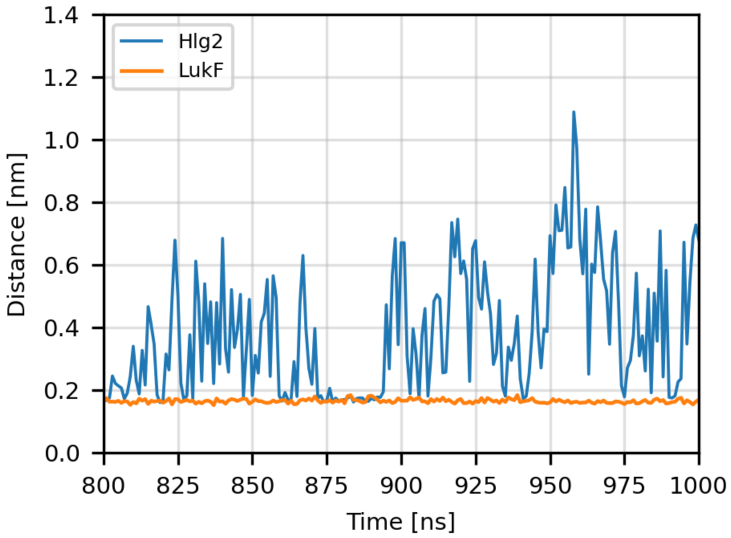

Figure S9: Minimum distance between each monomer and the membrane, in the last 200 ns of simulation. While LukF remains firmly bound to the membrane, Hlg2 forms only transient contacts with the lipids.

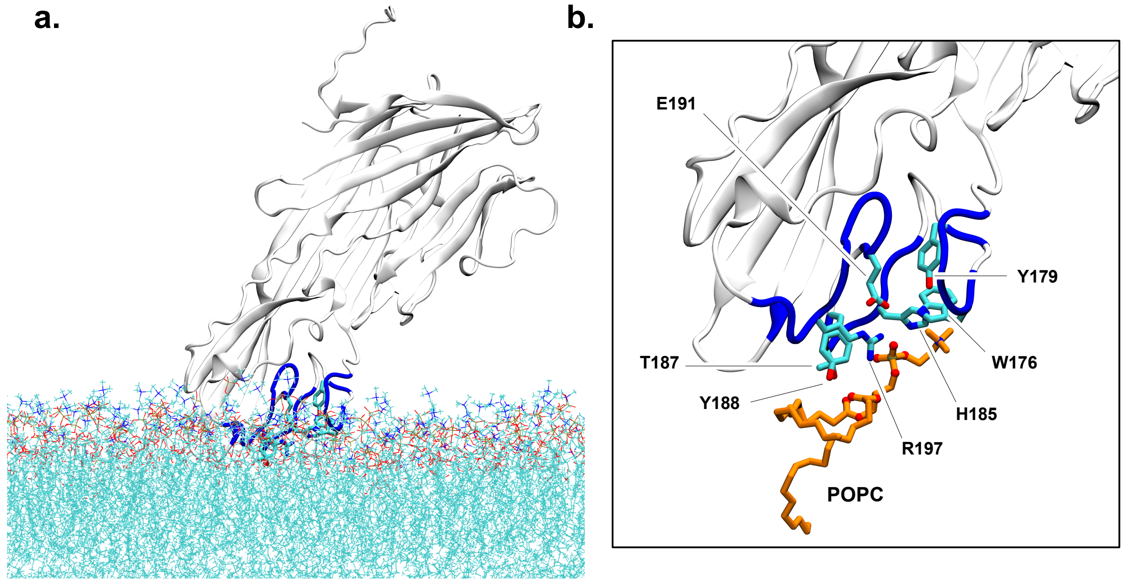

Figure S10: Interactions between LukF and the membrane, in the simulation capturing the spontaneous dimerization. **(a,b)** The residues forming the largest number of contacts with POPC molecules correspond to those highlighted in other studies, as described in the main text. Particularly evident is the coordination of a POPC molecule through a cation-π interaction involving W176 and the choline headgroup, and a salt bridge between R197 and the phosphate group of the lipid.

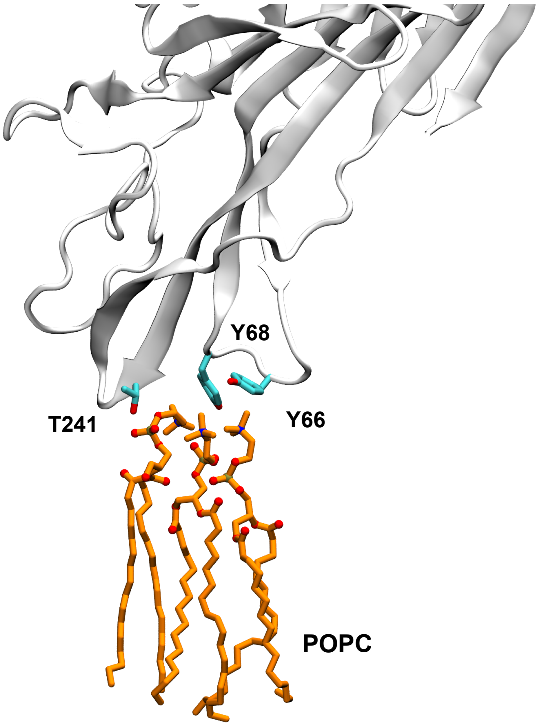

Figure S11: Transient interactions between LukF and the membrane. T241 interacts with the phosphate groups of POPC through electrostatic interactions, while Y66 and Y68 can both form electrostatic interactions through the hydroxyl group and cation-π interactions between the aromatic ring and the charged choline group.

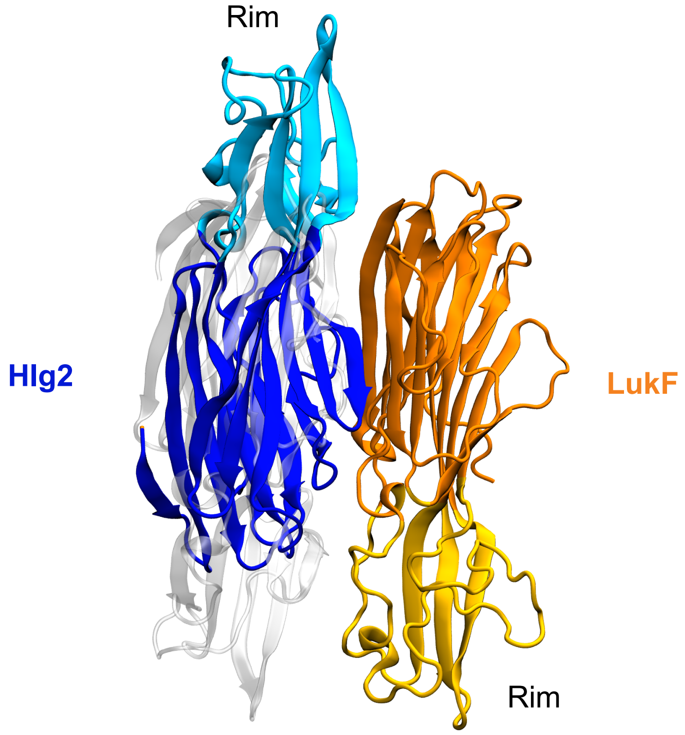

Figure S12: Representation of the best-score model obtained by docking Hlg2 and LukF without N-terminus. Contrary to the case where the N-ter is retained (semi-transparent cartoon representation), Hlg2 is here oriented in the opposite direction with respect to LukF, giving rise to what wee deem as "non-functional dimer". In particular, the rim domains, which are adjacent in the model obtained by spontaneous dimerization and in the structure of the final pore, are arranged in opposite directions one with respect to the other.

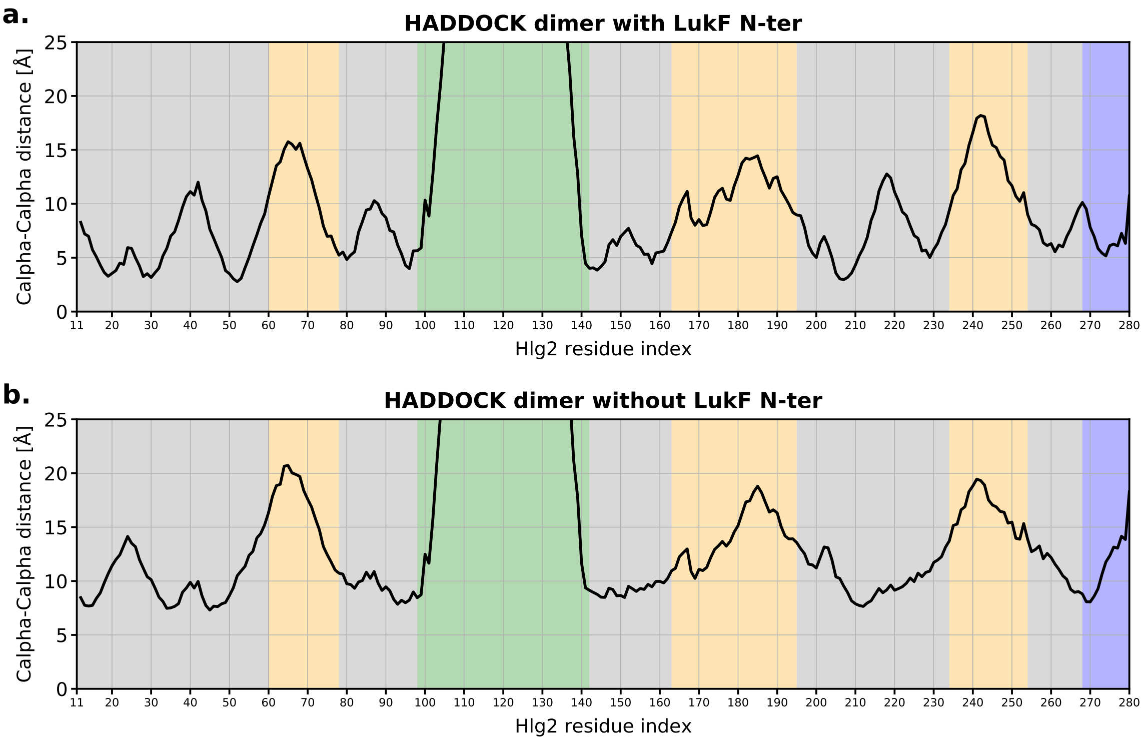

Figure S13: Difference between the C_α positions of Hlg2 in the HADDOCK model dimer and the same dimer in the crystal structure of the pore - as a function of the Hlg2 residue index - computed after a structural alignment made on the LukF monomers. The coloured areas indicate the different domains of the Hlg2 monomer: cap (gray), pre-stem (green), rim (orange), C-ter (blue). The plots report the values found for the dimers we selected from the pool of models predicted by HADDOCK **(a)** in the presence of the LukF N-ter (top-scored structure of cluster 2) and **(b)** in the absence of the LukF N-ter (top-scored structure of cluster 3).

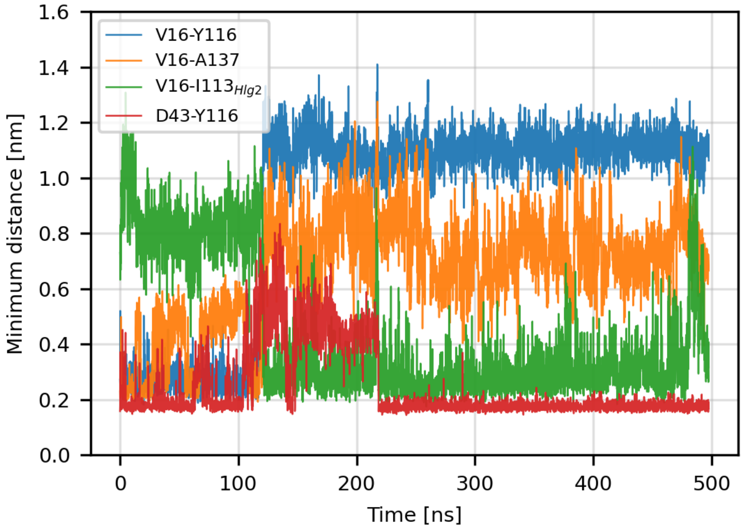

Figure S14: Minimum distance between residue V16 of LukF and other key residues, as monitored along the simulation of the HADDOCK dimer in the absence of LukF N-ter. All the contacts are between LukF residues, apart from the inter-molecular interaction between V16 of LukF and I113 of Hlg2.
